# Acute Kidney Injury in Hematopoietic Stem Cell Transplantation: A Review

**DOI:** 10.1155/2016/5163789

**Published:** 2016-11-03

**Authors:** Vinod Krishnappa, Mohit Gupta, Gurusidda Manu, Shivani Kwatra, Osei-Tutu Owusu, Rupesh Raina

**Affiliations:** ^1^Akron Nephrology Associates/Akron General Cleveland Clinic, Akron, OH, USA; ^2^Department of Internal Medicine, Akron General Cleveland Clinic, Akron, OH, USA; ^3^Onco-Hospitalist, Beth Israel Deaconness Medical Center, Boston, MA, USA; ^4^Department Hematology/Medical Oncology, Akron General Cleveland Clinic, Akron, OH, USA; ^5^Department of Nephrology/Internal Medicine, Akron General Cleveland Clinic, Akron, OH, USA

## Abstract

Hematopoietic stem cell transplantation (HSCT) is a highly effective treatment strategy for lymphoproliferative disorders and bone marrow failure states including aplastic anemia and thalassemia. However, its use has been limited by the increased treatment related complications, including acute kidney injury (AKI) with an incidence ranging from 20% to 73%. AKI after HSCT has been associated with an increased risk of mortality. The incidence of AKI reported in recipients of myeloablative allogeneic transplant is considerably higher in comparison to other subclasses mainly due to use of cyclosporine and development of graft-versus-host disease (GVHD) in allogeneic groups. Acute GVHD is by itself a major independent risk factor for the development of AKI in HSCT recipients. The other major risk factors are sepsis, nephrotoxic medications (amphotericin B, acyclovir, aminoglycosides, and cyclosporine), hepatic sinusoidal obstruction syndrome (SOS), thrombotic microangiopathy (TMA), marrow infusion toxicity, and tumor lysis syndrome. The mainstay of management of AKI in these patients is avoidance of risk factors contributing to AKI, including use of reduced intensity-conditioning regimen, close monitoring of nephrotoxic medications, and use of alternative antifungals for prophylaxis against infection. Also, early identification and effective management of sepsis, tumor lysis syndrome, marrow infusion toxicity, and hepatic SOS help in reducing the incidence of AKI in HSCT recipients.

## 1. Introduction

Hematopoietic stem cell transplantation (HSCT) has emerged as one of the most popular therapies for management of neoplastic disorders primarily lymphoproliferative disorders and bone marrow failure states including aplastic anemia and thalassemias [1, 2]. Every year, a number of transplants are conducted across the globe. However, the use of HSCT has been limited by an increased number of side effects noticed after the procedure is done. In this review, we will be discussing one of the major complications related to HSCT, namely, acute kidney injury (AKI).

Several different studies comparing the incidence of AKI amongst various HSCT modalities have been reported. Most patients undergoing HSCT develop AKI within the course of 1 year. However, the definition of AKI used has varied significantly amongst studies. Hence, the exact incidence of AKI is difficult to estimate. Currently, the incidence of AKI has been reported anywhere from 20% to 73% [3]. However, some studies have reported statistics as high as 92% [4].

## 2. Types of HSCT

Various types of HSCT are currently being used in today's era. These include myeloablative allogeneic, nonmyeloablative allogeneic, and autologous HSCT.

Myeloablative allogeneic HSCT involves use of a conditioning regimen composed of chemotherapy and radiation prior to the infusion of human leucocyte antigen matched donor cells [1, 5]. Conditioning regimens used in myeloablative HSCT involve cyclophosphamide, busulfan, cytarabine, and total body irradiation. Variations in the conditioning regimen have been reported amongst different studies especially depending on the malignancy [6, 7].

Nonmyeloablative allogeneic HSCT involves the use of a reduced intensity-conditioning regimen prior to the infusion of the donor cells. This modality of HSCT is better suited for patients with significant comorbidities [1]. Conditioning regimens involved in nonmyeloablative HSCT include fludarabine, busulfan, and cyclosporine [6, 8].

Autologous HSCT involves extraction of patient's own stem cells prior to administration of chemotherapy and radiation followed by infusion of the same stem cells after processing [3].

## 3. Prophylaxis against Graft-versus-Host Disease and Infections

All allogeneic transplant recipients receive prophylaxis against graft-versus-host-disease (GVHD). Regimens for prophylaxis include cyclosporine A (CsA), mycophenolate mofetil (MMF), tacrolimus (FK), or even short-term methotrexate (MTX) [9, 10]. However, the use of the above regimens for prophylaxis against GVHD is limited to allogeneic transplants. Furthermore, most of the patients discussed in various studies received prophylaxis against infections with acyclovir and azoles [11].

## 4. Treatment of Graft-versus-Host Disease

GVHD can be classified into acute and chronic categories based on duration of onset after HSCT and clinical findings. Acute GVHD has great significance as it can independently serve as a risk factor for the development of AKI in HSCT recipients [12]. The contribution of GVHD to AKI can be twofold. It could either be related to cytokine-mediated inflammation affecting the tubules/glomeruli or indirectly related to the use of medications such as cyclosporine, which by itself can predispose to nephrotoxicity. Furthermore, the presence of GVHD promotes viral reactivation such as cytomegalovirus (CMV), which can also contribute to AKI. The use of prednisolone in GVHD also predisposes to AKI [12]. Medical treatment options for GVHD include prednisone, antithymocyte globulin, sirolimus, and mycophenolate mofetil [3].

## 5. Definitions and Classification Systems of AKI Used across Various Studies

The incidence of AKI reported has varied significantly amongst various studies primarily due to the variations in definitions used for AKI (Table 1). In a recent study performed by Hingorani et al. in 2015, AKI was defined as a rise in serum creatinine of 0.3 mg/dL within 48 hours and/or 1.5 times the previous creatinine within 7 days [9]. In a study performed by Kang et al. in 2012, AKI following HSCT was defined as a rise in serum creatinine to 2-fold its initial value. Furthermore, severe AKI was defined as a rise in serum creatinine to the extent that it requires dialysis therapy [13]. In other studies performed by Yu et al. in 2010 and Caliskan et al. in 2006, AKI was graded on the basis of glomerular filtration rate (GFR) and requirement of dialysis, with grade 0 being a decrease in GFR to less than 25% of base value. Grade 1 was a fall of 25% or more in baseline GFR value and rise in serum creatinine less than 2-fold; grade 2 was a 2-fold or more increase in serum creatinine without requiring dialysis. Grade 3 on the other hand required dialysis with rise in serum creatinine to 2-fold or more [7, 14]. Prior to the above studies, analysis conducted by Zhou et al. and Lopes et al. defined AKI as doubling of serum creatinine in the first 100 days after transplantation [15, 16].

Acute Dialysis Quality Initiative (ADQI) group proposed AKI classification by the RIFLE criteria with R relating to risk, I to injury, F to failure, L to loss of kidney function, and E to end stage kidney disease. Three of these categories relate to severity, namely, risk, injury, and failure. Two of them relate to outcome, namely, loss and end stage kidney disease. AKI-R (risk) was defined as an increase in serum creatinine to 1.5 times base value or fall in GFR > 25% or decrease in urinary output to less than 0.5 mL/kg/h for 6 hrs. AKI-I (Injury) was defined as creatinine rise to 2 times the base value or fall in GFR > 50% or decrease in urine output to less than 0.5 mL/kg/h for 12 hrs. Furthermore, AKI-F (failure) was defined as rise in creatinine to 3 times the base value or fall in GFR by 75% or serum creatinine > 4 mg/dL or decrease in urine output to less than 0.3 mL/kg/h for 24 hrs or anuria for 12 hrs. Loss of renal functions was defined as total loss of kidney function requiring dialysis for >4 weeks and end stage renal disease was defined as requirement of dialysis for >3 months [17]. In 2007, the AKI network came out with a new staging system for AKI based on the previous RIFLE classification. It classified Stage 1 as rise in serum creatinine to >0.3 mg/dL or 1.5–2 times baseline value or fall in urine output to <0.5 mL/kg/h for >6 hrs, Stage 2 as rise in serum creatinine 2-3 times baseline value or fall in urine output to <0.5 mL/kg/h for >12 hrs, and Stage 3 as increase in serum creatinine > 3 times baseline value or fall in urine output to <0.3 mL/kg/h for 24 hrs or anuria for 12 hrs [18]. A comparative assessment study done by Ando et al. reflected that the AKI network criteria had lesser sensitivity in comparison to the RIFLE criteria to detect AKI in stem cell transplantation (SCT) patients especially in those with lower grades of AKI [6].

## 6. Incidence of AKI

Now that we have discussed the above variation in definitions of AKI, we will discuss the incidence of AKI reported across various studies for HSCT recipients. We will also analyze key differences in the incidence and risk factors of AKI amongst myeloablative allogeneic, nonmyeloablative allogeneic, and autologous transplant recipients (Table 2).

### 6.1. Myeloablative Allogeneic Transplant Recipients

The incidence of AKI reported in this subclass of transplant recipients is considerably higher in comparison to other subclasses such as nonmyeloablative allogeneic or autologous transplant recipients. Studies conducted by Ataei et al. in 2015 reported an incidence of AKI amongst myeloablative allogeneic recipients having acute myeloid leukemia (AML), acute lymphoblastic leukemia (ALL), and myelodysplastic syndrome (MDS) as 33% while using a conditioning regimen composed of busulfan and cyclophosphamide [19]. In a five-year study conducted by Kang et al. for patients receiving myeloablative allogeneic HSCT, incidence of AKI was reported at 18.8% [13]. However, some studies such as the one by Ando et al. have reported 66% incidence of AKI in patients receiving myeloablative allogeneic transplants [6]. Further more, a comparative study of AKI incidence between autologous and allogeneic transplant recipients revealed an incidence of 52% in the myeloablative autologous group versus 91% in the myeloablative allogeneic group [14].

Risk factors noted for the development of AKI in this subgroup include female sex, hypertension, sepsis, development of hepatic sinusoidal obstruction syndrome (SOS), GVHD, and use of amphotericin B. The conditioning regimen itself poses as a separate risk factor for the development of AKI [14, 19].

### 6.2. Nonmyeloablative Allogeneic Transplant Recipients

The incidence of AKI in this subclass is significantly lower in comparison to patients receiving myeloablative allogeneic transplants. In a multicenter retrospective study done by Liu et al. the incidence of AKI reported amongst patients receiving nonmyeloablative allogeneic stem cell transplantation (NMA-SCT) was reported as 29% with the majority of them developing AKI grade 1. The mortality rate was also found to significantly increase from AKI grade 1 (54.5%) to AKI grade 3 (71.4%) [5]. In a single center study conducted by Piñana et al. in Barcelona, 188 patients underwent reduced intensity-conditioning allogeneic HSCT and were analyzed for the incidence and risk factors contributing towards AKI. The incidence was reported at 52% with median time to development of AKI at 31 days. The majority of patients developed AKI grade 1 in comparison to grades 2-3 [8]. In a retrospective study conducted by Lopes et al. in Lisbon, Portugal, analyzing 82 patients receiving reduced intensity-conditioning allogeneic HSCT, the incidence of AKI by RIFLE criteria was reported up to 53.6% out of which 4.8% required dialysis [20].

Risk factors associated with the development of AKI in a study by Piñana et al. were diabetes mellitus, having received >3 prior lines of chemotherapy and use of MTX for prophylaxis against GVHD. The development of acute GVHD served as a risk factor only after 100 days had elapsed from the time of HSCT. Furthermore, no correlation was found between cyclosporine levels and the development of acute renal failure (ARF) [8]. Interestingly enough, diabetes mellitus plays a much bigger role in the development of ARF in these patients especially when combined with cyclosporine. Diabetic nephropathy by itself is one of the prime causes of end stage renal disease (ESRD). Furthermore, cyclosporine when used in conjunction can impair glucose metabolism by reducing insulin secretion and promoting resistance in the tissues to the peripheral effects of insulin [21]. Hence, cyclosporine use in diabetic patients, along with steroids which are used most often in this setting, has a synergistic effect on kidney injury especially within the first 100 days after HSCT [8]. On the contrary, another study showed that there was no association between diabetes mellitus and development of renal failure, and necessity of mechanical ventilation in NMA-SCT patients has been shown to be a strong risk factor for AKI development [22].

### 6.3. Myeloablative Autologous Transplant Recipients

The incidence of AKI in patients receiving myeloablative autologous transplants has ranged from 12% to as high as 52% [14, 16]. In a prospective study conducted by Caliskan et al. 47 patients undergoing HSCT were analyzed for the development of AKI. Comparison between autologous and allogeneic transplant recipients revealed an incidence of 52% in the autologous group versus 91% in the allogeneic group. Moreover, higher grades of AKI were reported in the allogeneic group compared to the autologous group. Risk factors associated with the development of AKI in the autologous group were sepsis, amphotericin B, and aminoglycoside nephrotoxicity. Sepsis and use of antibiotics and antifungals for sepsis contributed to 85% of renal dysfunction in the autologous study group. Cyclosporine and development of GVHD were major contributing factors to the development of AKI in the allogeneic group but neither of these was seen in the autologous group [14].

## 7. Pathophysiology of Acute Kidney Injury in HSCT

Multiple factors have been implicated in the causation of AKI after HSCT, which are discussed in detail including management options and have been summarized in Table 3.

### 7.1. Sepsis and Acute Kidney Injury

The contribution of sepsis to the development of AKI in patients receiving HSCT is manifold but studies have shown mixed results. Sepsis is more likely to occur in patients receiving HSCT generally due to immunosuppression owing to the prior malignancy or immunosuppressive therapy. The inflammatory response in sepsis leads to vasodilatation of the arterioles leading to decrease in effective intravascular volume being delivered to the kidneys [3]. This is more along the lines of a prerenal type of AKI. Furthermore, inflammatory mediators such as cytokines released during the initial phase are capable of damaging the renal tubules directly that results in a more renal type of AKI picture [23]. In a study by Merouani et al., sepsis and resulting hypotension accounted for increased AKI and higher mortality rates. Besides, hypotension was more prevalent in subjects with higher grades of renal failure [24]. On the contrary, in a study by Caliskan et al., hypotension failed to show positive correlation in the causation of AKI in autologous HSCT, although sepsis has been strongly implicated as a major factor in the development of AKI and higher mortality rates [14]. Another study found no association between sepsis and AKI but venoocclusive disease (VOC) and age have been shown to be risk factors for AKI in allogeneic HSCT [25]. Sepsis, use of nephrotoxic antibiotics for sepsis and hypotension may all have a synergistic effect resulting in AKI [3, 14].

### 7.2. Nephrotoxic Medications and Acute Kidney Injury

Several medications such as antifungals and antibiotics used in the treatment of sepsis, antivirals, and immunosuppressants, which are toxic to the kidney, may pose a direct risk and contribute significantly to the development of AKI.

*Amphotericin B* is one of the most commonly implicated agents for the development of AKI in HSCT recipients. It is capable of inducing renal vasoconstriction, hence hypoperfusion of the kidney resulting in tubular epithelium damage [3]. Certain preparations such as lipid based (liposomal) amphotericin have been studied and found to have a lower risk of kidney injury compared to conventional amphotericin [26, 27]. Nevertheless, a study by Hingorani et al. showed significantly increased risk of AKI associated with both conventional and liposomal amphotericin preparations and suggested to limit its use only for documented fungal infections or in situations where there has been usage of other antifungals for prophylaxis [28]. Alternately, newer antifungal drugs such as itraconazole, fluconazole, and voriconazole that are equally effective and have a better side effect profile should be substituted wherever possible [28, 29]. Recently, a study by Rocha et al. analyzed the relationship between urinary levels of neutrophil gelatinase-associated lipocalin (UNGAL) and amphotericin induced AKI. UNGAL is a potential early biomarker for drug induced AKI and found in higher levels in the amphotericin group with AKI than in subjects without AKI. This may serve as an early predictor of drug induced AKI and warrants employment of alternate options in advance to prevent deterioration of renal functions [30]. However, large-scale studies are needed to establish concrete evidence of this association.

*Antiviral medications such as acyclovir* are used in patients receiving HSCT and can contribute to the development of AKI. Acyclovir can precipitate in the renal tubules and collecting ducts forming crystals resulting in obstruction. This occurs especially with intravenous administration in high doses. Preexisting renal disease, rapid infusion of acyclovir, and dehydration predispose to the drug toxicity and development of AKI [31]. Most often AKI is asymptomatic but rarely presents with nausea, vomiting, and flank pain in less than 48 hours of acyclovir use and urinalysis may show hematuria and pyuria [32]. Polarizing microscopy of urinary sediment demonstrates birefringent needle-shaped crystals inside the leukocytes [33]. Management involves slower infusion, adequate hydration, and dosage modification in preexisting renal disease [31–33].

*Aminoglycosides* have also been implicated as an important cause for the development of AKI in HSCT recipients. No studies have clearly demonstrated aminoglycosides as an independent risk factor for the development of AKI; however, when used in sepsis or preexisting renal disease or in conjunction with other nephrotoxic medications, they do play a significant role. The toxicity of aminoglycosides is primarily due to intracellular accumulation of the drug that leads to alterations in cellular permeability in proximal renal tubules [3]; hence reduction in the frequency of dosing may help in reducing the incidence of nephrotoxicity. This hypothesis is supported by Olsen et al. through his study that once daily dosing of tobramycin even with higher doses in critical care patients reduced the incidence of nephrotoxicity. Furthermore, the same study found elevated urinary excretion of enzymes such as alanine aminopeptidase and N-acetyl-beta-D glucosaminidase in patients receiving multiple daily dosing compared to once daily dosing subjects. This increase in urinary enzyme level preceded elevation of serum creatinine and may serve as early markers of nephrotoxicity [34]. A meta-analysis of once daily dosing of aminoglycosides versus multiple daily dosing showed no difference in efficacies and toxicity rates between two regimens, although 2 of 22 studies revealed decreased risk of nephrotoxicity with once daily dosing [35]. Recently an update on current literature by Stankowicz et al. concluded that once daily dosing of aminoglycoside is therapeutically effective and minimizes the risk of drug toxicity and monitoring [36].

*Cyclosporine A* (CsA) is a calcineurin inhibitor used frequently for prevention of GVHD in HSCT recipients. Hence, the use of CsA is restricted to allogeneic transplant recipients [1, 3]. The mechanism by which cyclosporine use leads to renal damage is manifold and poorly understood. Calcineurin inhibitors are capable of causing renin angiotensin system activation and vasoconstriction of renal arterioles. They also decrease renal Klotho expression and increase oxidative stress causing renal endothelial damage [37]. Calcineurin inhibitors are also implicated in the pathogenesis of thrombotic microangiopathy (TMA), which serves as an independent risk factor for the development of AKI [38, 39]. Recently, a study demonstrated that calcineurin inhibitors such as tacrolimus and CsA augment production of renin and vascular endothelial growth factor (VEGF) in renal collecting ducts that causes renal ischemia and periductal fibrosis resulting in calcineurin-induced nephropathy. Furthermore, this process was attenuated by aliskiren, a direct renin inhibitor that may have clinical uses in the prevention of calcineurin-induced nephrotoxicity [40]. Another recent study showed that angiotensin receptor blocker, valsartan, has beneficial effects in preventing CsA related nephrotoxicity. Valsartan has been shown to reduce CsA induced oxidative stress and Klotho downregulation by unknown mechanisms, thereby decreasing the risk of nephrotoxicity [37].

Several studies have however failed to demonstrate a statistically significant relationship between blood CsA levels and development of AKI. For example, a retrospective study performed by Zhou et al. failed to demonstrate a relationship between CsA levels and development of AKI in a group of 86 patients undergoing allogeneic HSCT [15]. In another prospective study conducted by Piñana et al., where 188 patients undergoing reduced intensity-conditioning allogeneic HSCT were analyzed, CsA was implicated as the cause of ARF in 71% of patients, but no correlation was found between the blood levels of CsA and development of ARF [8]. In contrast, a study by Caliskan et al. showed CsA nephrotoxicity as the main factor in the causation of kidney failure, especially during 20–33 days after HSCT when CsA levels in blood starts declining. This indicates a time lag in the development of renal failure [14]. CsA can impair glucose metabolism and its use in diabetics may have a synergistic effect on kidney injury due to diabetes related nephropathy [8, 21]. Some studies in heart transplant recipients have demonstrated that replacing CsA by inhibitor of mammalian target of rapamycin such as sirolimus improves renal recovery; however further research is essential to assess its impact [41].

### 7.3. Hepatic Sinusoidal Obstruction Syndrome (SOS) and Acute Kidney Injury

Hepatic sinusoidal obstruction syndrome (SOS) previously known as venoocclusive disease (VOD) is a commonly encountered complication after HSCT and has been implicated to be an independent risk factor for the development of AKI. Although the pathophysiology of AKI in the SOS setting is poorly defined, it appears to be a variant of hepatorenal syndrome (HRS), which is, in itself, multifactorial in nature. HRS appears to be primarily hemodynamic and does not result in structural and renal lesions or tubular dysfunction [42].

Hepatic SOS is characterized by painful hepatomegaly, hyperbilirubinemia, fluid retention, and ascites. It is generally seen within 30 days of HSCT and the incidence widely varies ranging from 0 to 62.3% with a mean of 13.7% [43]. Hepatic SOS is more common in myeloablative allogeneic transplant recipients in comparison to others and exact pathogenesis remains unclear. However, the most favored theory is that sinusoidal endothelial cells and hepatocytes located within zone 3 of the acinus are damaged by the myeloablative conditioning regimen resulting in subendothelial deposition of fibrin and other blood products in the affected venules. This leads to progressive venular narrowing and obstruction that causes intrahepatic portal hypertension [44]. Furthermore, release of cytokines due to tissue injury and glutathione depletion due to chemotherapeutic drug detoxification by glutathione pathway causes hepatocellular necrosis and fibrosis [43, 44]. Reduction in nitric oxide has also been shown to correlate with the development of SOS due to disruption in the sinusoidal perfusion [45].

In a prospective study conducted by Moiseev et al., the increased number of circulating endothelial cells (CECs) was found to be associated with the incidence of hepatic SOS in patients undergoing allogeneic HSCT. On the day of SOS, the number of CECs was significantly higher than in the rest of the group. The cutoff level established for this assay was >100 CECs/mL and may serve as a biomarker marker for the diagnosis of hepatic SOS [46]. Studies have also established the utility of plasminogen activator inhibitor-1 (PAI) as a marker for the diagnosis and also for predicting the severity of SOS [47, 48].

Risk factors that predispose to development of hepatic SOS include presence of preexisting liver disease, use of methotrexate for prophylaxis against GVHD, and use of TBI > 12 cGy [49]. One of the most important risk factors related to the development of hepatic SOS is the preconditioning regimen. In a study conducted by Qiao et al., the effects of TBI and busulfan/cyclophosphamide were compared with regard to the incidence of hepatic SOS in mice. It was reported that both regimens damaged hepatic sinusoidal endothelial cells; however, the incidence of hepatic SOS was higher with busulfan-cyclophosphamide regimen compared to TBI [50]. Other risk factors include total parenteral nutrition for >1 week, young age (due to small sinusoidal lumens predisposing to easy occlusion), thalassemia major, osteopetrosis, and hemophagocytic lymphohistiocytosis in children; all have been shown to increase the risk of hepatic endothelial injury and SOS [51].

#### 7.3.1. SOS Diagnostic Criteria

Two different criteria have been established for the diagnosis of hepatic SOS, namely, the Seattle and Baltimore criteria. The Seattle criteria require the presence of at lease two of the three manifestations: jaundice, painful hepatomegaly, and fluid retention or weight gain within 20 days of HSCT [44, 52]. The Baltimore criteria require bilirubin of 2 mg/dL or greater plus two or more of these manifestations: painful hepatomegaly, weight gain > 5%, or ascites, within 21 days of HSCT [53].

#### 7.3.2. Grading of SOS

Severity of hepatic SOS is graded as mild when the illness fulfills the diagnostic criteria but is self-limiting without necessity of treatment, moderate when SOS subsides with diuretic or analgesic treatment, and severe when SOS persists beyond 100 days or results in death [44].

#### 7.3.3. Prevention of SOS

As conditioning regimens have been implicated as the primary risk factor, modification in the regimen has been found to reduce the risk of developing hepatic SOS. Oral busulfan when metabolized in the hepatic endothelial cells causes depletion of glutathione, hence promoting more oxidative stress and hepatic damage. Use of intravenous busulfan bypasses hepatic metabolism and has been found to reduce the risk of SOS development [54]. Use of MTX and tacrolimus for GVHD prophylaxis has been found to have a decreased incidence of SOS in comparison to tacrolimus, methotrexate, and sirolimus regimen [55].

There are several pharmacological measures that have been recently introduced for the management of hepatic SOS. Earlier studies implicated the use of tissue plasminogen activator (tPA) as well as methylprednisolone for short periods of time for the management of SOS. A study conducted by Yoon et al. established that the use of t-PA was associated with an increased risk of bleeding complications and the risk being greater with higher cumulative doses of tPA. Furthermore, it was advised against the use of tPA dose escalating protocols for management of moderate-severe SOS [56]. Recent studies have shown promising results with the use of defibrotide, a mixture of ss-oligodeoxyribonucleotides derived from porcine DNA, for the treatment of SOS. It acts by increasing the activity of tPA and inducing prostaglandin I_2_ and E_2_ production thereby preventing platelet aggression. Besides, defibrotide also has anti-inflammatory properties. Defibrotide has a greater beneficial effect if used within the first 2 days of HSCT [57]. A retrospective analysis conducted by Park et al. demonstrated the benefit of defibrotide for prophylaxis against SOS especially in high-risk patients [58]. More recently, phase 3 clinical trial concluded that defibrotide use is associated with significant survival benefit in hepatic SOS patients with multiorgan failure [59]. CART (Concentrated Ascites Reinfusion Therapy) is a novel technique involving the filtration, concentration, and reinfusion of drained ascites that has been shown to be a supportive care management option of tense ascites in SOS after HSCT [60].

### 7.4. Thrombotic Microangiopathy and Acute Kidney Injury

Thrombotic microangiopathy (TMA) such as thrombotic thrombocytopenic purpura (TTP) and hemolytic uremic syndrome (HUS) are not uncommon in HSCT, and the incidence has been reported anywhere from 0.5% to 63.6% [61]. TMA is primarily characterized by thrombocytopenia due to platelet aggregation, fragmentation of erythrocytes, and renal failure. However the cause of TMA in HSCT is different and is due to calcineurin inhibitors, total body irradiation (TBI), acute GVHD, and infections [38, 62]. The diagnosis of TMA in HSCT is often based on nonspecific laboratory features such as anemia, thrombocytopenia, and elevation in LDH and creatinine levels, which are commonly seen in patients receiving HSCT [62]. Hence, the true incidence of TMA may actually be underestimated. A retrospective study conducted by Changsirikulchai et al. on 314 patients who underwent hematopoietic cell transplant (HCT) revealed that development of acute GVHD grades II to IV, patient-donor sex mismatch, TBI > 1200 cGy, and adenovirus infections were risk factors for development of TMA. Further analysis revealed that cyclosporine did not contribute to the development of TMA [62]. On the contrary, studies done previously showed positive correlation between calcineurin inhibitor use and TMA in HSCT patients [38, 39]. The etiology of TMA in HSCT recipients was linked to endothelial injury either directly by GVHD or by cytokines released from GVHD occurring in other parts of the body [38, 62]. Also, some studies have revealed the role of VEGF in the pathogenesis of TMA and acute GVHD. Lower levels of VEGF were shown to be associated with severe forms of acute GVHD and TMA type glomerular injury [63, 64].

Management recommendations from the retrospective study were against adjustments in dosage or discontinuation of cyclosporine regimens, but favored continuing acute GVHD treatment. Plasma exchange is of limited use in treating HSCT associated TMA because the underlying etiology is not related to abnormal von Willebrand factor-cleaving protease. However, a case has been reported where CsA associated TMA and thrombocytopenia associated multiorgan failure were successfully treated with plasma exchange [65]. Eculizumab, a monoclonal antibody which prevents the production of membrane attack complex (MAC) by inhibiting conversion of C5 to C5a and C5b, has been successfully tried in the treatment of TMA secondary to calcineurin inhibitor and cytomegalovirus (CMV). Discontinuation of causative agents and early initiation of eculizumab therapy protects against further complement mediated host cell damage and also provides ample time for the treatment of infections [66, 67]. Recently, a retrospective study conducted by Arai et al. demonstrated that serum levels of neutrophil extracellular traps (NETs) were significantly elevated when compared to the pretransplantation level especially in patients developing TMA following HSCT. NETs may serve as an early biomarker for the detection of HSCT associated TMA [68].

### 7.5. Marrow Infusion Toxicity and Acute Kidney Injury

Marrow infusion toxicity has also been implicated in the pathogenesis of AKI amongst HSCT recipients. Patients can present with constellation of symptoms including hypotension, nausea, vomiting, and abdominal pain. Patients receiving harvested stem cells are often exposed to various cryoprecipitants such as dimethyl sulfoxide during the HSCT procedure. These toxic molecules are capable of causing hemolysis in the recipient resulting in precipitation of heme proteins in the distal tubule culminating in tubular obstruction [69]. A study demonstrated the presence of hemoglobin casts in renal tubules resulting in marked tubular dilatation and necrosis which was found at autopsy of patients who died after autologous bone marrow transplantation (BMT) [70]. Mainstays of management include alkalinization of urine, which increases heme solubility facilitating its excretion [71], and mannitol induced diuresis that prevents heme trapping in renal tubules [72].

### 7.6. Tumor Lysis Syndrome and Acute Kidney Injury

Anticancer therapy causes tumor cell lysis spilling intracellular contents in to the blood stream resulting in tumor lysis syndrome. As most patients undergoing HSCT are in remission at the time of transplantation, the incidence of tumor lysis contributing to AKI is very rare. However, certain malignancies, especially leukemias and lymphoproliferative disorders with high cell turnover, can undergo spontaneous tumor lysis [1, 3]. Newer chemotherapeutic regimens using flavopiridol to treat chronic lymphocytic leukemia (CLL) have been associated with a higher risk of tumor lysis syndrome [73]. Metabolic abnormalities in tumor lysis syndrome include hypocalcaemia, hyperphosphatemia, hyperuricemia, and hyperkalemia [74]. Renal injury is primarily due to precipitation of urate crystals and calcium phosphate in renal tubules [75]. Furthermore, hyperuricemia has been shown to be associated with crystal-independent renal damage by renal arteriolar vasoconstriction and release of proinflammatory cytokines that cause significant tubular injury [76]. The management of tumor lysis syndrome includes intravenous hydration and administration of agents such as rasburicase and allopurinol to reduce blood uric acid levels. Also, other management options are low phosphate diet and phosphate binders for hyperphosphatemia and renal replacement therapy for severe cases of AKI and resistant hyperkalemia [75].

### 7.7. Infectious Complications of HSCT and Acute Kidney Injury

Most patients after receiving HSCT are immunosuppressed and hence susceptible to a number of infectious complications. BK virus and adenovirus are common infections complicating HSCT, both of which are capable of inflicting damage to the kidneys.

*BK virus* is a member of the polyoma family and is generally seropositive in the majority of the population. Usually it is dormant in the urinary tract after primary infection in childhood; however, during times of immunosuppression, the virus reactivates. On reactivation, it causes damage to renal tubules and manifests either as nephritis or hemorrhagic cystitis with cystitis being the more common presentation [77]. The identification of BK virus infection is paramount in the course of the disease and most of the medications such as cidofovir, foscarnet, and leflunomide have not shown convincing data in the treatment. The major therapeutic option for BK virus nephritis is gradual reduction in immunosuppression. In a 5-year study by Hardinger et al., it was demonstrated that minimizing immunosuppression in patients with BK viremia improves graft survival and renal functions significantly [78]. Cystitis on the other hand resolves spontaneously and may only benefit from supportive care.

*Adenovirus* infection causes more commonly cystitis and rarely tubulointerstitial nephritis in HSCT recipients [79]. Adenovirus nephritis may manifest as fever, hematuria, and flank pain. Adenovirus cystitis has been reported to occur in a greater frequency especially amongst allogeneic transplant recipients and in patients with high grade GVHD and in those with severe immunosuppression [80, 81]. Management is supportive care but some case reports have proven that intravesical cidofivir therapy is superior to intravenous therapy against adenovirus cystitis; however, the data is very limited for now [82, 83].

## 8. Role of Albuminuria and Hypoalbuminemia in Acute Kidney Injury

Albuminuria has traditionally been defined as a urine albumin to urine creatinine ratio of 30 to 300 mg per gram of creatinine and has recently been identified in clinical studies to be a marker of endothelial damage in kidney and other organs [84]. In a prospective study conducted by Hingorani et al., 142 patients were analyzed for the development of albuminuria after receiving allogeneic and autologous HSCT. The incidence of albuminuria was more in patients with allogeneic HSCT compared to autologous HSCT. Albuminuria was found to be a useful marker of systemic inflammation and found to be associated with development of acute GVHD, hypertension, and progression of renal disease. However, this study did not establish any relationship between albuminuria and development of AKI [84].

Hypoalbuminemia, on the other hand, has a more significant relationship with the development of AKI in HSCT recipients. In a study by Caliskan et al., low baseline serum albumin levels correlated with the development of AKI. Possible explanation for this correlation is decreased oncotic pressure due to hypoalbuminemia results in intravascular volume contraction and renal ischemia. In addition, exposure to nephrotoxic drugs precipitates the condition. Hence, baseline serum albumin levels prior to HSCT may be an important risk factor for the development of AKI [14].

## 9. Novel Markers of Acute Kidney Injury in HSCT

One of the most traditional markers that have been used to establish the definition of AKI is serum creatinine. Although serum creatinine remains the gold standard, in this section we will discuss other biomarkers that have recently shown sensitivity for detection of kidney injury especially after HSCT. These may help guide us in the future to predict and treat AKI more aggressively.

*Elafin* is a protein that primarily produced by cellular elements such as epithelial cells and macrophages in response to injurious stimuli. It inhibits two key enzymes that mediate proteolysis, namely, proteinase 3 and neutrophil elastase. It has previously been identified as a marker in patients with GVHD. In a study conducted by Hingorani et al., increased urinary elafin levels were found to be associated with the development of albuminuria, AKI, progression to chronic kidney disease, and death in HSCT recipients [9].

*Urinary Liver-Type Fatty Acid Binding Protein (L-FABP)* was recently investigated as a marker for AKI after HSCT. In the study conducted by Shingai et al. involving 206 patients, the incidence of AKI was higher in patients with elevated baseline urinary L-FABP levels [85].

*Urinary Alpha 1M* is a urinary biomarker for predicting severe AKI. In a prospective study conducted by Morito et al., urinary alpha 1M was found to be useful for predicting severe AKI after HSCT [86].

## 10. Management of Acute Kidney Injury

AKI after HSCT has been found to be associated with an increased risk of mortality and decreased overall survival. Many studies have evaluated the utility of other therapeutic options for prevention of this complication. A recent randomized controlled trial conducted by Ataei et al. failed to demonstrate the benefit of N-acetyl cysteine in the prevention of AKI amongst myeloablative allogeneic transplant recipients [19]. Currently, avoidance of risk factors associated with the development of AKI remains the mainstay of management [3].

These would include the following:

- Use of the reduced intensity-conditioning regimen wherever possible
- Closer monitoring of nephrotoxic medications such as amphotericin or use of liposomal preparations
- Use of alternative antifungals such as fluconazole and voriconazole for prophylaxis against infection
- Early identification and management of sepsis
- Use of diuresis and alkalinization of urine in conditions such as tumor lysis syndrome or marrow infusion toxicity
- Early identification and management of hepatic SOS with defibrotide
- More importantly, early involvement of the nephrologist in the disease course is helpful in prevention of AKI and related complications.

## 11. Conclusion

One of the major limitations of HSCT in the management of lymphoproliferative disorders and bone marrow failure is AKI. The identified risk factors are GVHD, use of nephrotoxic medications, sepsis, hepatic SOS, tumor lysis syndrome, TMA, and marrow infusion toxicity. The management of AKI in HSCT recipients is primarily directed at prevention of risk factors that contributes to the development of AKI. This includes avoiding nephrotoxic medications wherever possible, use of reduced intensity-conditioning regimen, and early identification and management of sepsis, tumor lysis syndrome, hepatic SOS, and marrow infusion toxicity. Nevertheless, early involvement of the nephrologist in the disease process is most important for effective management of AKI and related complications.

## Figures and Tables

**Table 1:** Definitions and classification systems used for acute kidney injury.

Study/criteria	Classification	Definition
Hingorani et al. (2015)	AKI	Rise in Sr Cr of 0.3 mg/dL in <48 hrs and/or 1.5 times the previous level in <7 days

Kang et al. (2012)	AKI	Rise in Sr Cr to twice its initial value
	Severe AKI	Rise in Sr Cr to the level that requires dialysis

Yu et al. (2010) Caliskan et al. (2006)	Grade 0	Fall in GFR < 25% of the baseline value
	Grade 1	Fall in baseline GFR 25% or more and rise in Sr Cr < 2-fold
	Grade 2	More than or equal to 2-fold increase in Sr Cr without requiring dialysis
	Grade 3	More than or equal to 2-fold increase in Sr Cr requiring dialysis

Zhou et al. (2009) Lopes et al. (2006)	AKI	Doubling of Sr Cr in the first 100 days after transplantation

AKI network criteria (2007)	Stage 1	Rise in Sr Cr to >0.3 mg/dL or 1.5–2 times baseline value or fall in urine output to <0.5 mL/kg/h for >6 hrs
	Stage 2	Rise in Sr Cr 2-3 times baseline value or fall in urine output to <0.5 mL/kg/h for >12 hrs
	Stage 3	Rise in Sr Cr > 3 times baseline value or fall in urine output to <0.3 mL/kg/h for 24 hrs or anuria for 12 hrs

RIFLE criteria (2004) by ADQI group	AKI-R (risk)	Rise in Sr Cr 1.5 times baseline value or fall in GFR > 25% or fall in urine output to <0.5 mL/kg/h for 6 hrs
	AKI-I (injury)	Rise in Sr Cr 2 times the baseline value or fall in GFR > 50% or fall in urine output to <0.5 mL/kg/h for 12 hrs
	AKI-F (failure)	Rise in Sr Cr 3 times the baseline value or fall in GFR by 75% or Sr Cr > 4 mg/dL or fall in urine output to less than 0.3 mL/kg/h for 24 hrs or anuria for 12 hrs
	Persistent ARF	Total loss of kidney functions requiring dialysis > 4 weeks
	End stage renal disease	Requirement of dialysis for >3 months

AKI: acute kidney injury, Sr: serum, Cr: creatinine, GFR: glomerular filtration rate, ARF: acute renal failure, and ADQI: acute dialysis quality initiative.

**Table 2:** Incidence and risk factors for different types of HSCT.

Type of transplant	Incidence range	Risk factors
Myeloablative allogeneic transplantation	18.8–66%	Female sex, hypertension, sepsis, hepatic SOS, GVHD, amphotericin B

Nonmyeloablative allogeneic transplantation	29–53.6%	Diabetes mellitus, >3 prior lines of chemotherapy, acute GVHD, methotrexate use against GVHD prophylaxis

Myeloablative autologous transplantation	12–52%	Sepsis, amphotericin B toxicity, aminoglycoside (for sepsis) toxicity

HSCT: hematopoietic stem cell transplantation; Hepatic SOS: sinusoidal obstruction syndrome; GVHD: graft-versus-host disease.

**Table 3:** Pathophysiology and management of AKI in HSCT.

Etiology	Pathophysiology	Management/potential therapeutic options
*Sepsis*	Vasodilatation and reduced renal blood flow resulting in ischemia and direct renal tubular insult by inflammatory cytokines	Treatment of sepsis with appropriate medication

*Nephrotoxic medication*		
Amphotericin B	Vasoconstriction of renal vasculature resulting in hypoperfusion and renal tubular epithelial damage	Measurement of urinary UNGAL levels may serve as early biomarker of AKI. Restricting amphotericin use only for documented fungal infections. Use of antifungals with minimal nephrotoxicity such as itraconazole, fluconazole, and voriconazole.
		
Acyclovir	Formation of crystals in renal tubules and collecting ducts resulting in obstruction especially with IV administration in high doses	Demonstration of birefringent needle shaped crystals in urinary sediment under polarizing microscopy helps in diagnosis. Slower IV administration, hydration, and renal dose adjustments are recommended
		
Aminoglycosides	Intracellular accumulation in proximal tubules and change in cellular permeability	Measurement of alanine aminopeptidase and N-acetyl-beta-D glucosaminidase in urine may serve as an early biomarker of nephrotoxicity. Reduction in dosage frequency is the mainstay of management
		
Cyclosporine A	Renal vasoconstriction secondary to renin-angiotensin system activation. Increased production of VEGF. Downregulation of renal Klotho and increased oxidative stress causing renal endothelial damage. Thrombotic microangiopathy. Impaired glucose metabolism	Potential treatment options are aliskiren, valsartan, and switching to alternative immunosuppressant such as sirolimus

*Hepatic SOS*	Damage to hepatic sinusoidal endothelial cells by chemotherapeutic agents and subendothelial deposition of fibrin and other blood products resulting in venular obstruction. Glutathione depletion due to chemotherapeutic drug detoxification by glutathione pathway resulting in hepatocellular necrosis and fibrosis	Circulating endothelial cells (CECs) and plasminogen activator inhibitor-1 are potential biomarkers. Modification of conditioning regimens and use of defibrotide

*Thrombotic microangiopathy*	Renal endothelial injury by cytokines released in GVHD. Decreased levels of VEGF. Exposure to calcineurin inhibitors, TBI, and infections	Measurement of serum NETs level may serve as early biomarker for TMA. Continuing acute GVHD treatment may be of benefit. Plasma exchange has a limited role. Eculizumab may be a potential treatment option

*Marrow infusion toxicity*	Exposure to cryoprecipitants causes hemolysis and heme precipitation in distal renal tubules resulting in tubular obstruction	Alkalinization of urine and mannitol induced diuresis

*Tumor lysis syndrome*	Lysis of tumor cells releasing intracellular products into circulation resulting in hyperuricemia, hyperphosphatemia, hyperkalemia, and hypocalcemia. Precipitation of calcium phosphate and urate crystals causes damage to renal tubules. Vasoconstriction of renal arterioles and exposure to inflammatory cytokines causes injury to renal tubules	Mainstay of management involves IV hydration, rasburicase, and allopurinol. Low phosphate diet and phosphate binders for hyperphosphatemia. Medical management of hyperkalemia and renal replacement therapy in resistant cases and severe AKI

*Infections *		
BK virus	Immunosuppression reactivates dormant virus in urinary tract causing renal tubular injury and hemorrhagic cystitis	Reducing immunosuppression is the mainstay of treatment. Supportive care for cystitis.
Adenovirus	Tubulointerstitial nephritis and cystitis	Supportive care. Intravesical cidofivir is potential treatment option for cystitis

UNGAL: urinary neutrophil gelatinase-associated lipocalin; AKI: acute kidney injury; IV: intravenous; GVHD: graft-versus-host disease; VEGF: vascular endothelial growth factor; TBI: total body irradiation; TMA: thrombotic microangiopathy; SOS: sinusoidal obstruction syndrome; NETs: neutrophil extracellular traps.
